# Inactivation of face-selective neurons alters eye movements when free viewing faces

**DOI:** 10.1073/pnas.2309906121

**Published:** 2024-01-10

**Authors:** Reza Azadi, Emily Lopez, Jessica Taubert, Amanda Patterson, Arash Afraz

**Affiliations:** ^a^Unit on Neurons, Circuits and Behavior, Laboratory of Neuropsychology, National Institute of Mental Health, NIH, Bethesda, MD 20892; ^b^Section on Neurocircuitry, Laboratory of Brain and Cognition, National Institute of Mental Health, NIH, Bethesda, MD 20892; ^c^School of Psychology, The University of Queensland, Brisbane, QLD 4072, Australia

**Keywords:** eye movement, inferior temporal cortex, face processing, macaque

## Abstract

During free viewing, faces attract gaze and induce specific fixation patterns corresponding to the facial features. This suggests that neurons encoding the facial features are in the causal chain that steers the eyes. However, there is no physiological evidence to support a mechanistic link between face-encoding neurons in high-level visual areas and the oculomotor system. In this study, we targeted the middle face patches of the inferior temporal (IT) cortex in two macaque monkeys using an functional magnetic resonance imaging (fMRI) localizer. We then utilized muscimol microinjection to unilaterally suppress IT neural activity inside and outside the face patches and recorded eye movements while the animals free viewing natural scenes. Inactivation of the face-selective neurons altered the pattern of eye movements on faces: The monkeys found faces in the scene but neglected the eye contralateral to the inactivation hemisphere. These findings reveal the causal contribution of the high-level visual cortex in eye movements.

## Significance

It has been shown, for more than half a century, that eye movements follow distinctive patterns when free viewing faces. This suggests causal involvement of the face-encoding visual neurons in the eye movements. However, the literature is scant of evidence for this possibility and has focused mostly on the link between low-level image saliency and eye movements. Here, we bring causal evidence showing how face-selective neurons in the inferior temporal cortex inform and steer eye movements when free viewing faces.

In the retina, high concentration of photoreceptors can be found only in the fovea; thus, the most efficient way for the visual system to gather information is to move the eyes to survey the visual field. Unguided scanning of the visual field is inefficient; thus, the oculomotor system needs to rely on the incoming visual information to steer the eyes. As a result, a bidirectional causal motif forms: The input to the eyes guides the eye movements, which in turn select the input to the eyes.

How does the visual input direct eye movements? Saliency maps have been proposed to predict the location of future fixations by weighting the features in a scene according to their visual saliency (1). Earlier models of saliency maps relied solely on low-level visual properties such as brightness, contrast, and spatial frequency (2–4). However, high-level visual properties such as complex object features also affect eye movement behavior. Therefore, recent saliency map models have begun to incorporate complex features to improve their predictive power (5–9). These models suggest functional connectivity between parts of the brain that process high-level object information and the oculomotor system. Nevertheless, there is currently no neurophysiological evidence supporting this idea.

Complex object features are processed in high-level visual areas such as the inferior temporal (IT) cortex (10–12). Therefore, we hypothesized that neural activity in the IT cortex should play a causal role in controlling eye movements. To investigate this hypothesis, we chose to study the link between eye movements when free viewing faces and face-selective neurons in the IT cortex (13, 14). There are a number of reasons for this choice. First, determining the preferred stimulus of a group of cells in the IT cortex is less speculative for the case of face-selective neurons. Second, these neurons are typically clustered, making it possible to perturb the activity of a group of cells with similar function (13, 15). Third, faces have geometrical regularities that facilitate quantitative analysis of the results (16). Fourth, the causal role of face-selective neurons in visual perception is well established in the literature (15, 17, 18). Last but not least, the natural pattern of eye movements when viewing faces is relatively standard and distinct from other objects and thus can be easily quantified and tracked.

The specific patterns of eye movements during free viewing of faces were first noticed by Yarbus (19) and consistently reported since (20–23). In “eye movement and vision” (19), Yarbus describes: “... the faces of the people shown in the picture attract the observer’s attention much more than the figures …” and then later “... when looking at a human face, an observer usually pays most attention to the eyes, the lips, and the nose.” This pattern of eye movement has also been reported in nonhuman primates (24), and we will refer to it as Yarbus T (25).

Low-level saliency models may partially explain this behavior because eyes and mouth typically have higher visual contrast (26), nevertheless, possible involvement of high-level visual processing in steering the eye movements while viewing faces and complex objects cannot be rejected.

**Fig. 1.**
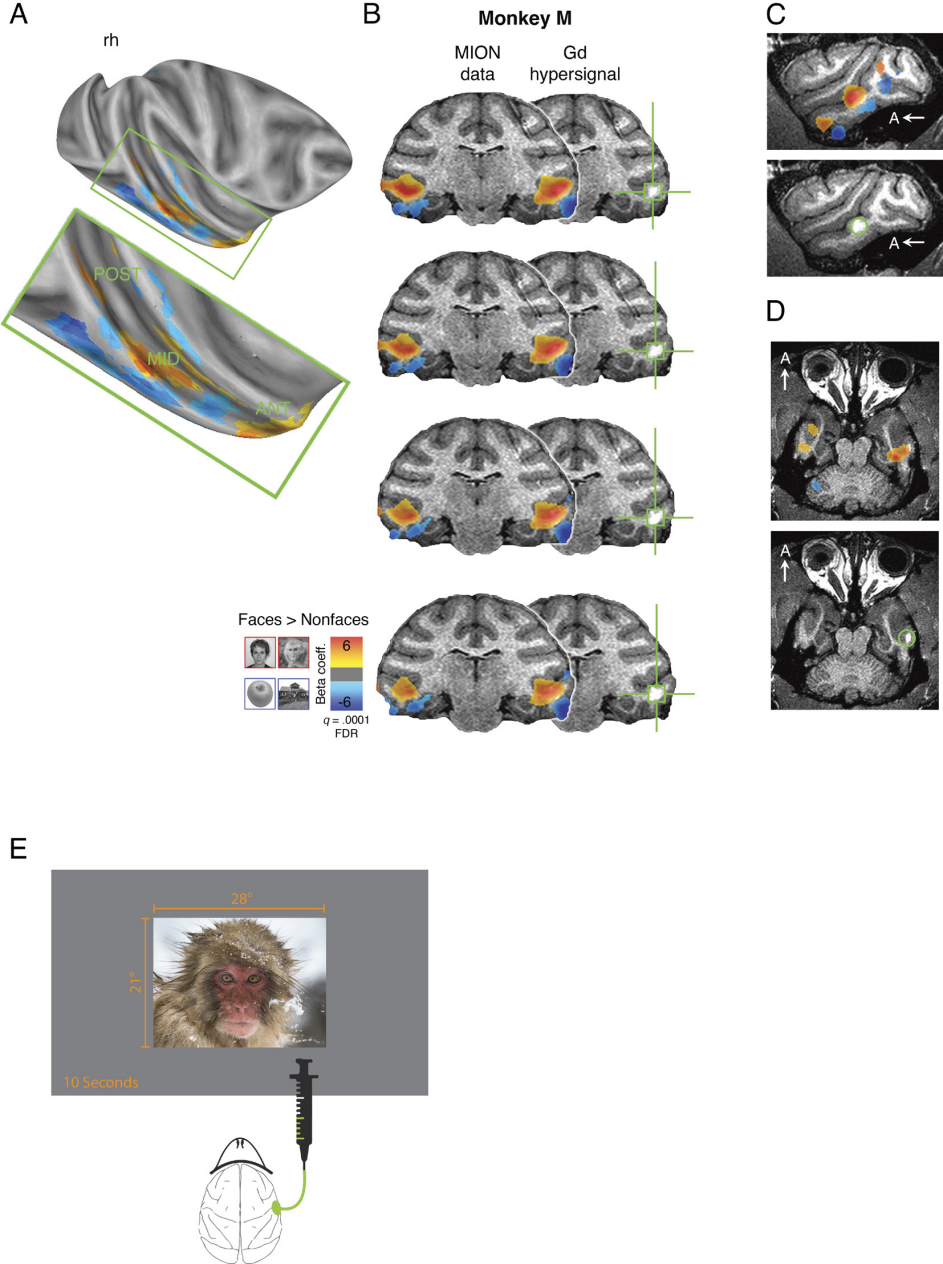
Study overview. (*A*) Functional localizer data from Monkey Mr projected onto a partially inflated cortical surface, showing a lateral view of the *Right* hemisphere. The voxelwise statistical threshold was set at q = 0.001 (corrected for false discovery rate). The localization procedure successfully identified multiple face-selective patches, primarily in the posterior (POST), middle (MID), and anterior (ANT) regions of the superior temporal sulcus. (*B*–*D*) Post-gadolinium-diethylenetriamine pentaacetic acid (Gd-DTPA) infusion anatomical scans with Beta maps superimposed, illustrating the contrast between face stimuli (hot colors) and nonface stimuli (cool colors) to validate the targeting procedure. The bright white hypersignal is associated with the infusion. (*B*) Coronal slices with 1 mm separation, starting approximately +2 mm anterior to the interaural line and presented posterior to anterior (*Top* to *Bottom*). The green crosshairs indicate the signal from injected Gd-DTPA. (*C* and *D*) Sagittal and transverse views. A = anterior. (*E*) Monkeys’ eye movement was recorded during free viewing of visual stimuli presented on a screen subtending 28° × 21° during baseline sessions and after injection of 5 or 10 µL in the middle face patch of the *Left* or the *Right* hemisphere.

Here, we aimed at testing the role of IT face-selective neurons in eye movements by causal manipulation of their function. We used muscimol, a strong GABA_A_ agonist (27) and potent neural silencer, to reversibly inactivate clusters of face-selective neurons, as well as control regions unilaterally in the IT cortex of two macaque monkeys (Fig. 1). We then investigated the resulting effects on eye movements when free viewing faces and other objects. We chose to target the middle face patch in the face processing network because it contains the largest number of face-selective neurons, it can be reliably localized, and it is hypothesized to be homologous to the human fusiform face area (13, 28–32). Face-selective cells retain some retinotopy and are shown to causally contribute to face recognition behavior only in their contralateral visual field (17); thus, we chose to target only one hemisphere at a time. Using this design, we can compare the behavioral results across the two hemifields within each injection session, a feature that increases the statistical power of the study. Moreover, given the logistic limitations of performing microinjection in a deep brain structure like the IT cortex (*Methods*), unilateral injections are much safer for the animal.

## Results

In this experiment, two male macaque monkeys (*Macaca mulatta*) were used. Each trial began with the animals fixating on a central fixation point for 1 s before an image (28 × 21 dva) was displayed on the screen. The image was chosen randomly from a set of 62 images, consisting of human and nonhuman primate faces, objects, plants, and scenes. The image was displayed for 10 s on the screen. During the image presentation, the animals were not required to hold fixation; instead, they could freely move their eyes and were rewarded for keeping their gaze within the image boundaries every 1 to 3.5 s. We used an fMRI face/object localizer to locate the middle face patch in the IT cortex (13, 33, 34) (Fig. 1 *A*−*D*), as well as control areas with no face selectivity, for muscimol microinjections. These microinjections were performed through a cranial chamber. We also recorded the animals’ natural behavior without any injection in multiple separate baseline sessions interleaved with injection sessions. We employed two different volumes of muscimol: 5 and 10 µL, which were injected into the middle face patch and control regions in separate injection sessions.

### Natural Viewing Patterns for Faces.

**Fig. 2.**
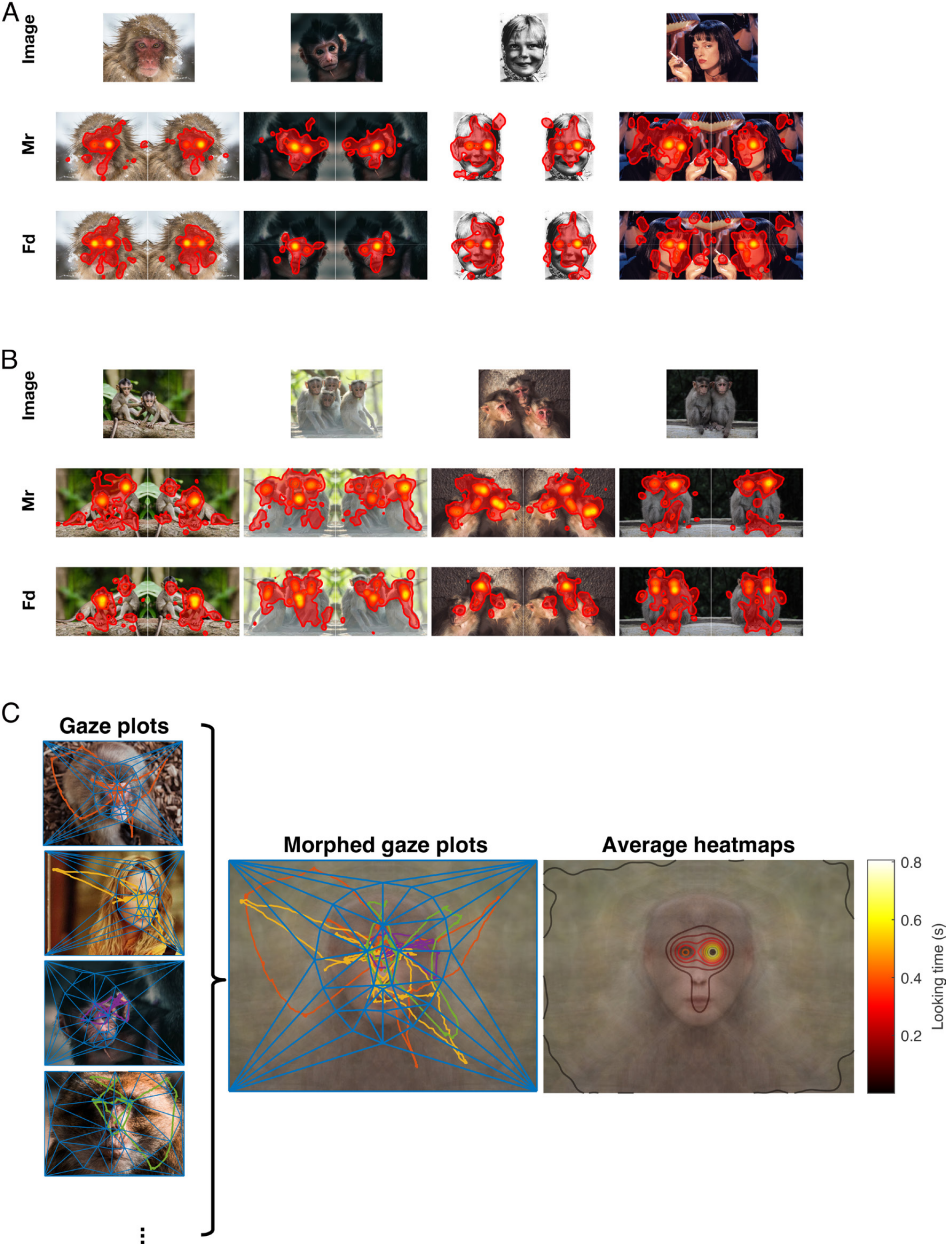
Eye movement patterns for visual stimuli containing faces in baseline sessions. (*A* and *B*) Gaze heatmaps were generated to illustrate the typical eye movement patterns of monkeys Mr and Fd during baseline trials for images with a single face (*A*) and multiple faces (*B*). Heatmaps represent fixation gaze positions for each image revealing that the monkeys primarily directed their gaze toward the faces and exhibited a strong focus on the eyes for both human and nonhuman primate faces. (*C*) Morphing was used to average gaze positions and heatmaps across multiple trials. The *Left* column depicts gaze positions for a few exemplar trials in which a face was presented. The blue lines indicate the triangulation on corresponding points for each face. The *Middle* panel illustrates an average face created by warping and averaging all the faces observed by the monkeys, along with the corresponding gaze positions morphed using the same affine transformation matrix. The *Right* panel displays the average gaze heatmaps for all baseline trials presented to monkey Mr, demonstrating the Yarbus-T effect. The results showed a bias toward fixating on the right eye.

First, we aimed at systematic documentation of the monkeys’ natural free viewing patterns for the faces in our study. Fig. 2 provides examples of average fixation heatmaps on both monkey and human faces across all baseline trials. During these trials, the animals consistently identified and fixated on faces, doing so in 98.9 and 98.7% of the trials for monkeys Mr and Fd, respectively, with a reaction time of only 695 and 418 ms. Additionally, our results indicated that the majority of the looking time in each trial was on faces, accounting for 68.1 and 72.1% of the total trial time for monkeys Mr and Fd, respectively. This establishes that similar to human observers, monkeys prefer to spend more time looking at faces.

Does the fixation pattern follow the Yarbus T? Systematic investigation of gaze patterns offers a technical challenge; how to compare patterns of eye movement across multiple faces? To study this within the entire category of primate faces (including human, ape, and monkey faces), we employed face morphing techniques that utilize corresponding points to generate an average gaze heatmap, by merging the data from all trials. First, we identified the corresponding points on each face to construct an average primate face shape. We then used triangulation to determine the affine transform matrix, which was applied to warp gaze positions (*Methods*). An average gaze heatmap was computed across all trials in which a face was displayed (Fig. 2*C*). Analysis of the average gaze heatmap from baseline trials revealed that the monkeys primarily directed their gaze toward the interior of the presented faces, with particular emphasis on the eyes, nose, and mouth. However, both monkeys exhibited a noticeable preference for the right side of the faces. This baseline bias was characterized by the animals spending significantly more time looking at the right eye compared with the left side. This resulted in 1.1 and 0.1 s difference in fixation times for monkeys Mr and Fd respectively (permutation test with 10,000 resamples; *P* < 0.001 for both animals). These results are consistent with earlier findings in macaque monkeys (24). Throughout the course of this study, we did not observe any systematic change in baseline bias (*SI Appendix*, Fig. S1). To accurately account for fluctuations in baseline bias, we normalized the data from injection sessions using baseline biases recorded within a month prior and after.

### The eFfect of Neural Inactivation on Free Viewing of Faces.

**Fig. 3.**
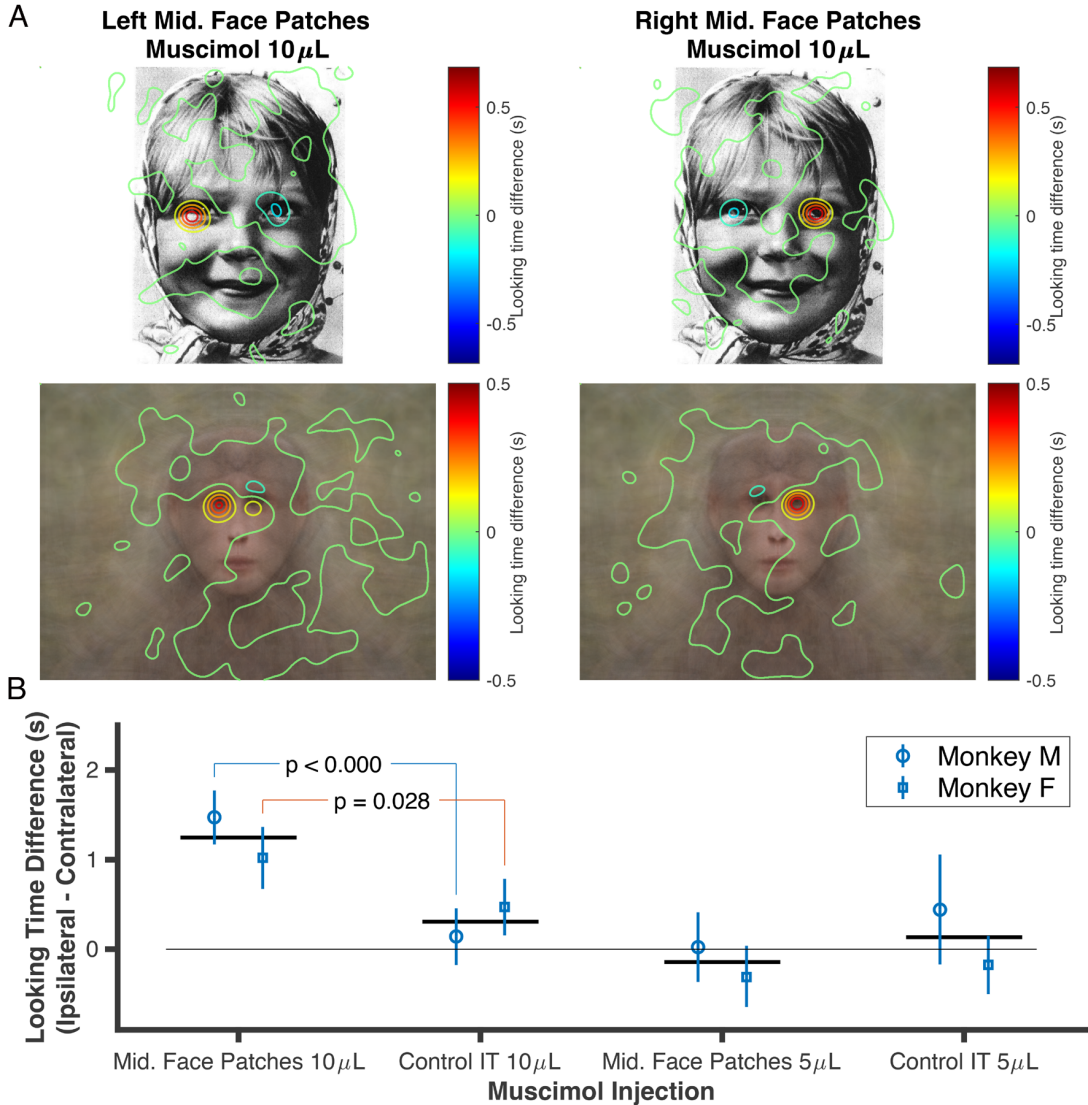
The effect of unilateral neural inactivation in the IT cortex on eye movement traces during viewing of faces. The animals exhibited a relative increased duration of gaze toward the eye ipsilateral to the muscimol injection side. (*A*) The average change in the fixation pattern for a typical face image (*Top*) and average of morphed faces (*Bottom*) after 10 µL muscimol injections in the *Left* and *Right Middle* face patches for monkey Mr. The heatmaps indicate a greater focus on the ipsilateral eye (with respect to the injection side) compared to the contralateral one. (*B*) The difference in looking time for the ipsilateral and contralateral eye under different injection conditions for each animal. The error bars represent the 95% CI, and the horizontal solid lines indicate the average across the animals.

After injecting 10 µL of muscimol in the middle face patch, the monkeys developed a strong preference for looking at the ipsilateral eye compared to the one contralateral to the injection hemisphere. Fig. 3*A* demonstrates changes in the gaze heatmap for a typical image for monkey Mr, highlighting a greater fixation on the ipsilateral side of the face relative to the contralateral side. This trend is also evident in the average gaze heatmaps gathered from all trials featuring primate faces on the screen (Fig. 3). To statistically analyze this finding, we defined a rectangular area around each eye on the average face and calculated the time that the animals looked at each eye. We then compared the difference in time spent fixating on each eye. The results showed that after injection of 10 µL of muscimol in the middle face patch, the monkeys on average spent 1.4 and 1.0 s more on the ipsilateral eye (permutation test with 10,000 resamples; *P* < 0.001 and < 0.001; respectively for monkey Mr and Fd). Injection of 10 µL of muscimol in the control area created a similar but weaker effect for monkey Fd and no effect for Monkey Mr (0.1 and 0.4 s looking time difference; permutation test with 10,000 resamples; *P* = 0.519 and 0.008 respectively for monkeys Mr and Fd). Analysis of the 5 µL condition did not show any significant level of change in fixation (permutation test with 10,000 resamples; *P* = 0.097 to 0.908 for all comparisons. All the *P*-values calculated by testing differences in looking times are corrected using the Benjamini–Hochberg procedure). Moreover, the effect of 10 µL muscimol in the control area for monkey Fd was significantly weaker than the same volume of muscimol in the face patch (permutation test with 10,000 resamples; *P* = 0.028).

Despite muscimol injections altering the pattern of eye movement within the facial frame, faces remained the most salient visual stimulus. Throughout the trials, the animals consistently spent the majority of their time looking at faces, ranging from 96.5 to 100% of the total looking time for Monkey Mr, and 97.2 to 100% for Fd. The results indicated no significant differences between the baseline and postinjection trials (permutation test with 10,000 resamples; *P* > 0.288 and 0.217, for all injection conditions compared to the baseline, respectively for Monkey Mr and Fd). Moreover, muscimol injections did not affect the latency of the initial saccade toward faces, which ranged from 534 to 717 ms for monkey MR, and 347 to 577 ms for Monkey Fd (permutation test with 10,000 resamples; *P* > 0.267 and 0.086 for comparison of all injection conditions compared with baseline for Monkey Mr and Fd). These findings demonstrate that the animals exhibited no difficulty in locating and fixating on the faces presented on the screen.

### The Effects on Free Viewing Nonface Objects.

Analyzing the potential effects of IT inactivation on looking patterns for nonface objects presents a technical challenge. It is not possible to define corresponding points on nonface objects thus, unlike faces, we cannot morph them into an average form. Therefore, to investigate the potential effect of IT inactivation on nonface objects, we analyzed eye movement on each image separately. This takes a toll on the statistical power of the analysis as we cannot pool the results for nonface objects. Moreover, the existence of Yarbus T for faces allows performing hypothesis testing in search of a potential change of eye movement pattern as shown earlier. However, this is not the case for nonface objects, which leaves only the possibility of searching for a general change in gaze patterns. Given these two limitations, it is not possible to firmly reject a possible effect on looking patterns for nonface objects, even though we did not find any evidence for it.

**Fig. 4.**
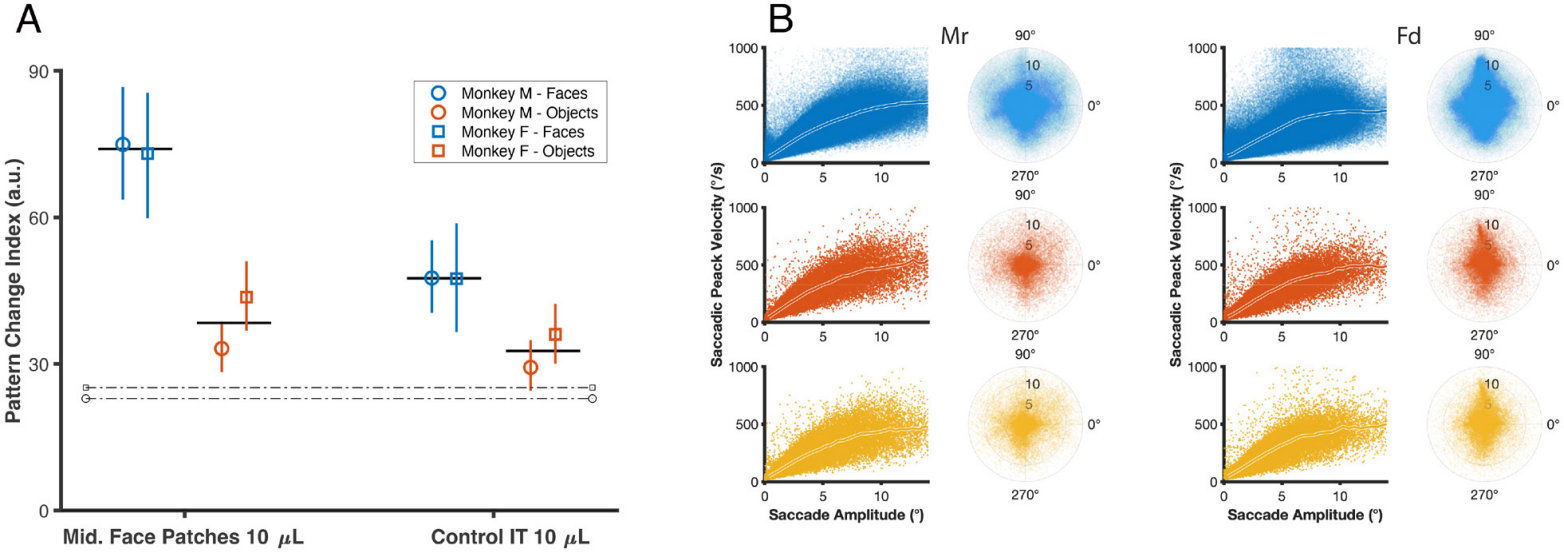
Pattern change index and saccadic properties before and after muscimol injections. (*A*) This plot illustrates the pattern change index across injection conditions. The error bars indicate the 95% CI, and the solid horizontal lines represent the average across the animals. The dashed bars represent the expected natural variation in the patterns of eye movements estimated from baseline sessions. (*B*) Saccade properties remained unaffected following muscimol injection. Main sequence and saccade landing points are illustrated for baseline conditions (blue), as well as 10 µL muscimol injections in the *Left* (red) and *Right* (yellow) *Middle* face patches for both monkeys in trials with a face image. No significant differences were observed between the main sequences and saccade landing points after muscimol injection.

To conduct statistical analysis on changes in gaze heatmaps after muscimol injections, we computed a pattern change index. This index is the normalized variance of Euclidean distance between the gaze patterns of baseline and postinjection trials for each image (see *Methods* section for more details). Fig. 4*A* displays the average pattern change index for each condition. Our results indicate that the pattern change index was the highest for faces following injection, and we observed a significant difference between the face images when muscimol was injected into the middle face patches (permutation test with 10,000 resamples, *P* < 0.001 for all comparisons).

### The Effects on Motor Features of Saccades.

To assess the impact of muscimol injections on motor features of saccadic behavior, we examined certain general saccade properties acquired from baseline and 10 µL middle face patch trials. Fig. 4*B* shows the main sequence and polar plot of saccadic landing points for both baseline and sessions conducted after the administration of muscimol in the middle face patch. A regression analysis (*Methods*) showed that the saccadic peak velocity followed the main sequence patterns after muscimol injections in the face patches (*R*^2^ = 0.72, 0.68 for monkey Mr and 0.69, 0.69 for monkey Fd, respectively, for right and left hemispheres). This finding indicates that the oculomotor system retains its capacity to plan and execute saccades effectively.

## Discussion

In line with Yarbus predictions, the present results revealed that faces are behaviorally salient stimuli for the visual system. Morphing the gaze patterns on multiple faces allows us to confirm that free viewing gaze patterns of monkeys naturally follow the Yarbus T. Furthermore, inactivation of face-selective neurons in the IT cortex alters the patterns of eye movements during free viewing of faces. This intervention does not affect the ability to saccade to faces in the periphery. However, when looking directly at faces, inactivating face-selective neurons causes the animals to spend more time viewing the eye ipsilateral to the inactivated side; trimming the contralateral branch of the Yarbus T. These findings also indicate that inactivation of similar volumes of the IT cortex outside the face-selective area and inactivation of smaller volumes of the cortex (inside or outside the face area) do not induce a comparable effect. We did not observe any specific changes in viewing patterns over nonface objects, although we cannot reject this possibility and we will further discuss it later in this section.

These results indicate that the activity of IT face-selective neurons is a part of the causal chain for eye movement control. But what does it mean to be part of a causal chain? The notion of causality has been used in different ways in systems neuroscience. The most strong form of causality, referred to as “causal production” (35), is when the mere occurrence of an event is enough to produce another event. However, the causal contribution of IT face-selective units to eye movements is not causal production in that it is not a direct motor command. Instead, the causal role of face-selective neurons is rather shaping the saliency profile that steers the gaze. This form of causality, also known as “causal dependence,” implies that the activity of face-selective cells is among the multiple factors that contribute to eye movements. Nevertheless, this contribution is causal (as opposed to correlational) because experimental randomization of one factor affects the other (36).

Are the observed effects consistent with the physiological response properties of IT neurons? Early studies indicate that IT neurons, including face-selective units, often have large bilateral receptive fields, but typically biased toward the contralateral field, even as wide as ~30° (11, 37). However, recent studies have refined and quantified these findings. Notably, the size of IT receptive field can vary depending on the measurement approach (38), with more recent studies reporting sizes as small as 2.5° of visual angle (39, 40). Nevertheless, there is general agreement that typical IT receptive fields are foveally biased, and most IT neurons are less responsive to far peripheral stimuli. Furthermore, IT responses are not bilaterally symmetric and most of the drive comes from the contralateral visual field (39). More importantly, the causal contribution of IT face-selective cells is limited to the stimuli presented in the contralateral hemifield (17), at least for the case of a face discrimination task. In sum, IT receptive fields are not global and exhibit a bias toward the contralateral visual field. This is consistent with the neglect-like effects observed in our study.

**Fig. 5.**
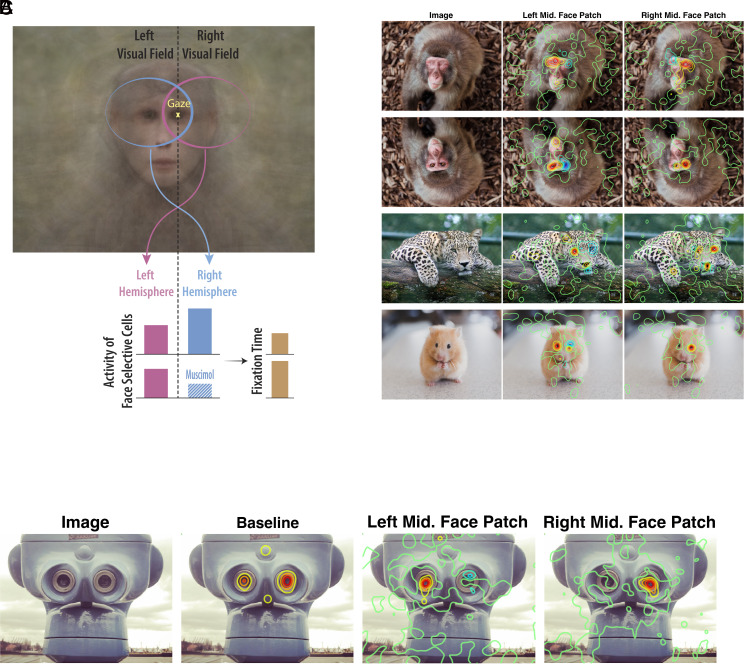
A schematic framework for understanding the effects and a few examples of boundary conditions. (*A*) We do not have neural recording data available in this study; this framework is merely a schematic, provided based on what is known about neural receptive fields in the IT cortex to put the observed effects in neural context. Given the foveal and contralateral biases of IT neurons, it is expected that during fixation on the right eye (represented by the yellow “x”), the receptive fields of face-selective neurons in the *Right* hemisphere encompass both eyes. In contrast, the *Left* hemisphere’s receptive fields include only one eye, fewer facial features, and a smaller part of the face. Consequently, this configuration is expected to result in increased activity levels in the face-selective neurons of the *Right* hemisphere. Muscimol injection would suppress these activities and invert the balance, ultimately leading to a prolonged fixation time. (*B*) Average changes in eye movement patterns are provided for examples of boundary condition images for monkey Mr. (*C*) Average baseline and change in eye movement patterns are presented for face pareidolia for monkey Mr.

Considering the topographical properties of IT receptive fields, we can estimate the activity of face-selective neurons when fixating on an eye of a face. To enhance understanding, a schematic is provided in Fig. 5*A* to illustrate this estimated activity when the monkey fixates on the right eye (with respect to the observer) of a face. In this gaze arrangement, the foveated eye (right) falls within the receptive field of face-selective neurons in both hemispheres, while the other eye (left) and most of the facial features are only present in the receptive field of the right hemisphere face cells. Therefore, it is reasonable to assume that face-selective neurons of the right hemisphere exhibit more activity when the gaze is directed at the right eye, and vice versa. At this point, we appeal to an analogy from low-level visual areas to put the inactivation results in context. The primary visual cortex contains neurons that respond to salient features, and activation of these neurons attracts the gaze toward their receptive fields. Now, consider a subregion of the primary visual cortex inactivated by muscimol. A salient visual stimulus presented in the corresponding visual field would be ignored, and fail to elicit any eye movements. Similarly, an eye is a highly salient stimulus activating the face-selective neurons in the IT cortex and their activity attracts the gaze toward their receptive field. As shown in Fig. 5*A*, unilateral muscimol injections diminish the activity of the face-selective neurons representing the saliency of an eye presented in the ipsilateral visual field, resulting in reducing the time spent on the contralateral side. We present this framework here solely to help organize the current findings. However, direct evaluation of this framework requires follow-up studies, including systematic recording of the neural activity in the absence of inactivation, as well as microstimulation of face-selective neurons during free viewing of faces.

How does inactivation of face neurons alter the gaze patterns for direct viewing but not for finding the faces in the periphery? We did not inactivate all of the face neurons even within a hemisphere, and it is possible that the activity of the remaining units is sufficient for the search for faces in the periphery. Alternatively, it is possible that the mechanisms underlying finding faces in the periphery and viewing the features within faces are separate from each other. According to this possibility, low-level saliency of faces presented in the periphery is enough to attract the gaze. Experimental rejection of the first scenario requires inactivation of all face neurons. Nevertheless, performing multiple and prolonged injections offers a technical challenge due to safety considerations and this experiment has to wait for more nimble techniques of neural inactivation. One possible candidate for this purpose is the use of Designer Receptors Exclusively Activated by Designer Drugs (DREADD)-based chemogenetic tools (41, 42) where virus injections can be performed in separate sessions but they can all be activated simultaneously.

An interesting study by Sadagopan et al. (43) demonstrated that monkeys’ face detection performance during free viewing is indeed affected when the mediolateral face patch is inactivated by muscimol. This apparent discrepancy with our findings can be attributed to two factors: First, eye movements were not measured in this study, leaving open the possibility that the eye movements toward faces were unaffected, while the animals’ perceptual face detection was impaired. Second, the visibility of the faces used in that study was modified down to near detection threshold, whereas the faces that we employed were in full contrast, concealing a potential subtle effect. Further investigation is required to differentiate between these possibilities. Another study by Roy et al. (44) revealed that reversible inactivation of the posterior superior temporal sulcus (rich in face neurons) impairs gaze following behavior in primates, where they move their eyes to follow the gaze of other primates. While gaze following is not directly related to face detection, the causal role of face-selective neurons in gaze following provides an additional causal link between the function of face-selective cells and eye movements.

To explore the boundary conditions, we added a few altered images to the image set including upside-down faces and faces without eyes or with the eyes placed out of the facial frame. Even though this was not the central focus of the current study, we decided to report some of these anecdotal observations as they may inform future experiments. Fig. 5*B* illustrates the effect of inactivation of face-selective units on some of the altered images after injection of 10 µL of muscimol in the face patch. In the case of inverted face condition, we were curious to see whether there is a similar lateralized effect and if so, which side of the face would be ignored. An object-centered representation of faces (45, 46) predicts flipping the effect side, in contrast, a head-centered representation (47–49) anticipates persistence of the effect on the same side with respect to the observer. The results were consistent with a head-centered basis for cortical representation of faces. The other image alteration tested here was to eliminate the eyes on a monkey face and cover their place with face skin. This alteration was inspired by two facts. First, our effects seem to be mainly focused on the eyes of a viewed face. Second, previous studies suggest that the eyes are the main driver of the majority of face-selective units in the posterior face patch (50) as well as the anterior medial face patch (51). Looking patterns for these unusually altered faces elicited comparable effects to unaltered faces; the animals spent more time looking at where the ipsilateral eye was supposed to be and ignored the corresponding contralateral position. This shows that an “eye” is not defined for the face processing system merely by its internal features such as a round pupil and that contextual cues can also inform the system (52).

Another boundary condition tested here was the case of face pareidolia. The phenomenon of perceiving faces in objects, known as face pareidolia, is not limited to humans; it also happens to rhesus monkeys (53). Fig. 5*C*, the bottom row, depicts a case of such a phenomenon, a tower-viewer that resembles a face. Consistent with Taubert et al. (53) the monkeys’ eye movement patterns resembled a Yarbus T where the binoculars of the tower-viewer corresponded to the eyes of a face. Is this pareidolia effect mediated through the face-selective neurons? While correlational involvement of face-selective parts of the cortex in face pareidolia has been shown (54), our results establish a causal link between IT face-selective units and face pareidolia.

Is IT activity generally used to drive eye movements or is it a special case for faces? Our findings did not indicate any systematic change in eye movement patterns for objects following muscimol injection in the IT cortex. However, we cannot reject the possibility of such changes entirely. Unlike faces, objects lack corresponding points, making it impractical to systematically aggregate gaze heatmaps across multiple objects. Consequently, the statistical power to detect a significant effect in the case of objects is diminished. Moreover, given the clustering of face-selective neurons in the cortex, muscimol would likely affect neurons with similar properties, augmenting the behavioral impact of inactivation. As a result, the causal role of other IT neurons in eye movements for nonface objects remains a possibility for further exploration using more specific techniques. For instance, molecular labeling and perturbation of object-selective neuronal populations that are not spatially clustered (55–60) can follow-up this question.

In conclusion, the findings presented in this study establish a causal relationship between the activity of face-selective neurons in the IT cortex and the oculomotor system. These results indicate how high-level visual processing can determine the future input to the visual system by steering the eyes toward the relevant parts of the visual field. They also highlight the potential utility of eye movements as a valuable tool for investigating visual processing within high-level visual areas.

## Methods

In this study, we collected free viewing eye movement data from two adult male rhesus monkeys (*M. mulatta*), designated as Mr and Fd. All procedures were carried out in accordance with the guidelines of the National Institute of Mental Health Animal Use and Care Committee.

### Functional Localization.

#### Data acquisition.

To identify the discrete regions or patches of the IT cortex that responded preferentially to faces, we used an independent functional localizer experiment (61–65). While the animals were awake and fixating, we presented images from six different categories including human faces, monkey faces, scenes, objects, phase scrambled human faces, and phase scrambled monkey faces. Each category contained 30 grayscale cropped images presented on a 12 dva square canvas. All six categories were presented in each run in a standard on/off block design creating 12 blocks in total. lEach ‘stimulus on’ block consisted of presenting 15 images sequentially, each displayed for 900 ms, followed by a 200 ms interstimulus interval, resulting in blocks lasting 16.5 s. The arrangement of blocks and the sequence of images within each block were randomized. The images were displayed using Psychtoolbox (66, 67) and the PLDAPS toolbox (68) in MATLAB (MathWorks, version R2018b). Eye position was monitored and recorded using a magnetoresistance-compatible infrared camera (MRC Systems, Heidelberg, Germany) at 60 Hz. The animals received juice rewards, every 0.6 to 3 s, as long as they maintained their gaze within a 4 dva diameter central fixation window. Runs were excluded from the analysis if the monkey's gaze did not remain within the fixation window for more than 70% of the time.

Functional data were acquired in a 4.7T Bruker Biospin scanner with a vertical bore (Bruker Biospec, Ettlingen, Germany). Before each scanning session, a contrast agent (monocrystalline iron oxide nanocolloid; MION) was injected into the femoral vein to increase the hemodynamic response. MION doses were determined independently for each animal (~8 to 10 mg/kg). We collected whole-brain images with a four-channel transmit-and-receive radiofrequency coil system (Rapid MR International). A low-resolution anatomical scan was also acquired in the same session to serve as an anatomical reference [modified driven equilibrium Fourier transform (MDEFT) sequence: voxel size, 1.5 by 0.5 by 0.5 mm; field of view (FOV), 96 by 48 mm; matrix size, 192 × 96; echo time (TE), 3.95 ms; and repetition time (TR), 11.25 ms]. Functional echo planar imaging (EPI) scans were collected as 42 sagittal slices with an in-plane resolution of 1.5 by 1.5 mm and a slice thickness of 1.5 mm. The TR was 2.2 s, and the TE was 16 ms (FOV = 96 × 54 mm; matrix size, 64 × 36 m; flip angle, 75°). For anatomical registration and MR targeting, we also acquired high-resolution T1-weighted whole-brain anatomical scans under sedation in a 4.7T Bruker scanner with an MDEFT sequence. Imaging parameters were as follows: voxel size, 0.5 by 0.5 by 0.5 mm; TE, 4.9 ms; TR, 13.6 ms; and flip angle, 14°. These scans were used to create a high-resolution template for each animal.

#### fMRI preprocessing and localization.

All EPI data were analyzed using Analysis of Functional NeuroImages (AFNI) software (http://afni.nimh.nih.gov/afni) (69). Raw images were first converted from Bruker into AFNI data file format. The data collected in a single session were corrected for static magnetic field inhomogeneities using the PLACE algorithm (70). The time series data were then slice time–corrected and realigned to the volume with the minimum outliers. All the data for a given animal were aligned to the corresponding high-resolution template for that animal, allowing for the combination of data across multiple sessions. The first two volumes of data in each EPI sequence were discarded. The volume-registered data were then spatially smoothed with a 3-mm Gaussian kernel and rescaled to reflect percentage signal change from baseline. The statistical significance values of the differential signal changes from the functional scans were projected onto the high-resolution anatomical scan and surface model for visualization. The face-selective patches in ITC were identified in all animals using the following contrast: activations evoked by (human faces + monkey faces) > activations evoked by (scenes + objects; see Fig. 1 *A*–*D*). We targeted the mediolateral face patches based on the corresponding peak activations in the middle superior temporal sulcus. The targeted control areas were chosen on the convexity of the IT cortex 4 to 5.5 mm anterior and 4 to 8 mm ventral to the middle face patches where no face selectivity was observed.

### Free Viewing Experiment.

#### Apparatus.

The experiments were conducted in a dark room using NIMH MonkeyLogic (71) on MATLAB (MathWorks, version R2019a). The animals were positioned 57 cm away from a 32-inch, 1,920 × 1,080 pixel, 120 Hz, LCD Monitor (Display++, Cambridge Research System). Eye tracking was performed using monocular corneal reflection and pupil tracking (Eyelink 1000 Plus, SR Research) at a frequency of 1,000 Hz.

#### Behavioral experiments.

Each session began with a calibration procedure consisting of 13 points at the start of each session. The monkeys initiated a trial by fixating for 1 s on a central fixation point (a black circle with a radius of 0.3°) against a gray background. An image (28° × 21°) was then displayed on the screen for 10 s. The monkeys received liquid reward every 1 to 3.5 s as long as they maintained their gaze on the image. Any breaks in looking at the image that lasted less than 500 ms were ignored, allowing the animals to blink naturally.

Only the trails that the animals spent more than 7 s were included for further analysis. The image was randomly selected from a set of 62 unique images, along with their left-right flipped versions, making a total of 124 images. The animals performed 124 and 78 baseline sessions, and 28 and 23 muscimol injection sessions respectively for monkey Mr and Fd. Table 1 xyz illustrates the number of injections on each site and volume for each monkey. On average, the animals performed 135.7 and 321.1 trials in each session respectively for monkey Mr and Fd.

**Table 1.** Number of injection sessions for each condition in each animal

	Face patches 10 µL		Control 10 µL		Face patches 5 µL		Control 5 µL	
	Left	Right	Left	Right	Left	Right	Left	Right	Total
Monkey Mr	4	4	5	6	3	3	2	1	28
Monkey Fd	3	3	3	3	4	2	3	2	23

#### Injection apparatus and parameters.

For microinjections, a handmade injection circuit was used, based on the design presented by Noudoost and Moore (72); a 30-gauge (0.304 μm outer diameter) stainless steel lancet point cannula with a long 15° primary bevel, coupled with a 5 to 25 µL gas-tight syringe (1700 series, Hamilton), was used for drug delivery. For infusion, we used a syringe pump (Pump 11 Elite, Harvard Apparatus). For cannula insertion and extraction, a 3d-printed grid and an oil hydraulic micromanipulator (MO-952, Narishige) were used for accurate targeting and minimal damage to the cortex. To prepare the muscimol solution, a commonly used GABA_A_ agonist, muscimol powder (Sigma-Aldrich) was diluted in normal saline to a concentration of 5 mg/mL and then filtered through a sterile filter.

Injections were performed in both the left and right hemispheres through a rectangular cranial chamber measuring 45 × 25 mm (coronal × sagittal dimensions). The chamber was centered at the sagittal midline and approximately 8 mm anterior–posterior stereotaxic coordinate. During the high-resolution T1-weighted whole-brain anatomical scan, the cranial chambers were filled with a clinical-grade MR contrast agent gadolinium-diethylenetriamine pentaacetic acid (Gd-DTPA), which was diluted with normal saline to 5 mmol/L. This enabled us to identify the four corners of the grid positioned within the chamber. We then selected the appropriate grid hole and depth for each target position based on the angle of grid holes 30° by fitting a plane on the four corners.

To confirm the accuracy of the targeting procedure, we infused 1 µL Gd-DTPA diluted with normal saline to 5 mmol/L. The cortical spread of Gd-DTPA is similar to that of muscimol, as shown in previous studies (73), and can be detected in an anatomical MRI scan. We assessed the overlap of Gd-DTPA spread with the functionally defined peak activations by aligning the postinjection anatomical volume and functional localizer data in a post hoc analysis (Fig. 1 *B*–*D*).

#### Saccade detection.

To detect saccades, we applied a velocity threshold criterion of six SD from the median saccade velocity during fixation at the beginning of each trial. The start and end points of saccades were determined by marking the points where velocity fell below two SD from the median saccade velocity during fixation.

#### Fixation heatmaps.

To generate the fixation heatmaps, we excluded saccades greater than 1 dva from the eye movement traces. Next, we superimposed all the eye movement traces from each trial onto a heatmap grid. We then applied a 2d Gaussian filter to the heatmaps, with a SD of 0.25 dva.

#### Morphing.

To average facial features and analyze eye movement patterns on the primate faces presented during the experiment, we start by manually identifying 26 corresponding points on each face. The average face shape is then determined by calculating the mean x and y coordinates for each corresponding point. To create corresponding triangles on each face, we use the Delaunay Triangulation.

For each triangle, we can create an affine transform using the following equation:

$$\left[\begin{array}{ccc} x_{1}^{\prime } & x_{2}^{\prime } & x_{3}^{\prime }\\ y_{1}^{\prime } & y_{2}^{\prime } & y_{3}^{\prime }\\ 1 & 1 & 1 \end{array}\right]=T_{i}\left[\begin{array}{ccc} x_{1} & x_{2} & x_{3}\\ y_{1} & y_{2} & y_{3}\\ 1 & 1 & 1 \end{array}\right],$$

where *xs* and *ys* represent the vertices of a triangle on the original image, and *x*’s and *y*’s represent the vertices for the corresponding triangle on the morphed image. *T*_i_ is the affine transform matrix for triangle i that can be calculated using the above formula. This allows us to transform any point (*x, y*) from the original image within the triangle *i* to new coordinates on the new image using the following equation:

$$\left[\begin{array}{c} x^{\prime }\\ y^{\prime }\\ 1 \end{array}\right]=T_{i}\left[\begin{array}{c} x\\ y\\ 1 \end{array}\right].$$

#### Variance of gaze heatmaps after injection.

To check the effect of injection, we calculated the variance of gaze heatmaps before and after injection. For this, we resampled heatmaps to 140 × 105 pixels (providing 5 pixels per degree in visual angle) and then applied a 2d Gaussian convolution with a SD of 2.5 pixels. Then, for each trial, we defined a pattern change index:

$$\frac{d\left(h_{i},\bar{\bar{H}}\right)-d\left(h_{i},\bar{h}\right)}{d\left(h_{i},\bar{\bar{H}}\right)},$$

where d is the Euclidean distance between two heatmaps, *h*_i_ is gaze heatmap for trial *i*, $\bar{h}$ is the average gaze heatmap for the visual stimulus across all trials in injection condition, and $\bar{\bar{H}}$ is the grand average of gaze heatmaps across all the trials in the injection condition and all the baseline trials for the same visual stimulus. The Euclidean distance between two heatmaps (e.g., *u* and *v*) can be calculated by the following equation:

$$d\left(u,v\right)=\sqrt{\sum_{i\;=\;1}^{n}{\left(u_{i}-v_{i}\right)}^{2}}.$$

#### Analysis of saccadic main sequence.

We employed regression analysis to fit a polynomial function to saccadic properties derived from trials in baseline sessions and each injection condition, treating each animal separately. The polynomial function used was as follows:

$$PV=\beta \sqrt{A}.$$

Here, *PV* represents the saccadic peak velocity, *A* denotes the saccadic amplitude, and *β* is the regression coefficient. The regression analysis yielded substantial results, with R-squared values ranging from 0.65 to 0.72 for monkey Mr and 0.56 to 0.69 for monkey Fd.

## Supplementary Material

Appendix 01 (PDF)

## Data Availability

All data and codes referenced in this article, whether in the main text or *SI Appendix*, are accessible on Zenodo at https://doi.org/10.5281/zenodo.10426591 (74).
