# Supplementary material for: Inactivation of face-selective neurons alters eye movements when free viewing faces
Source: Proc Natl Acad Sci U S A. 2024 Jan 10;121(3):e2309906121. doi: 10.1073/pnas.2309906121 (PMC10801883; doi:10.1073/pnas.2309906121)
Supplement: Supplementary file 1 — Appendix 01 (PDF) [file pnas.2309906121.sapp.pdf]

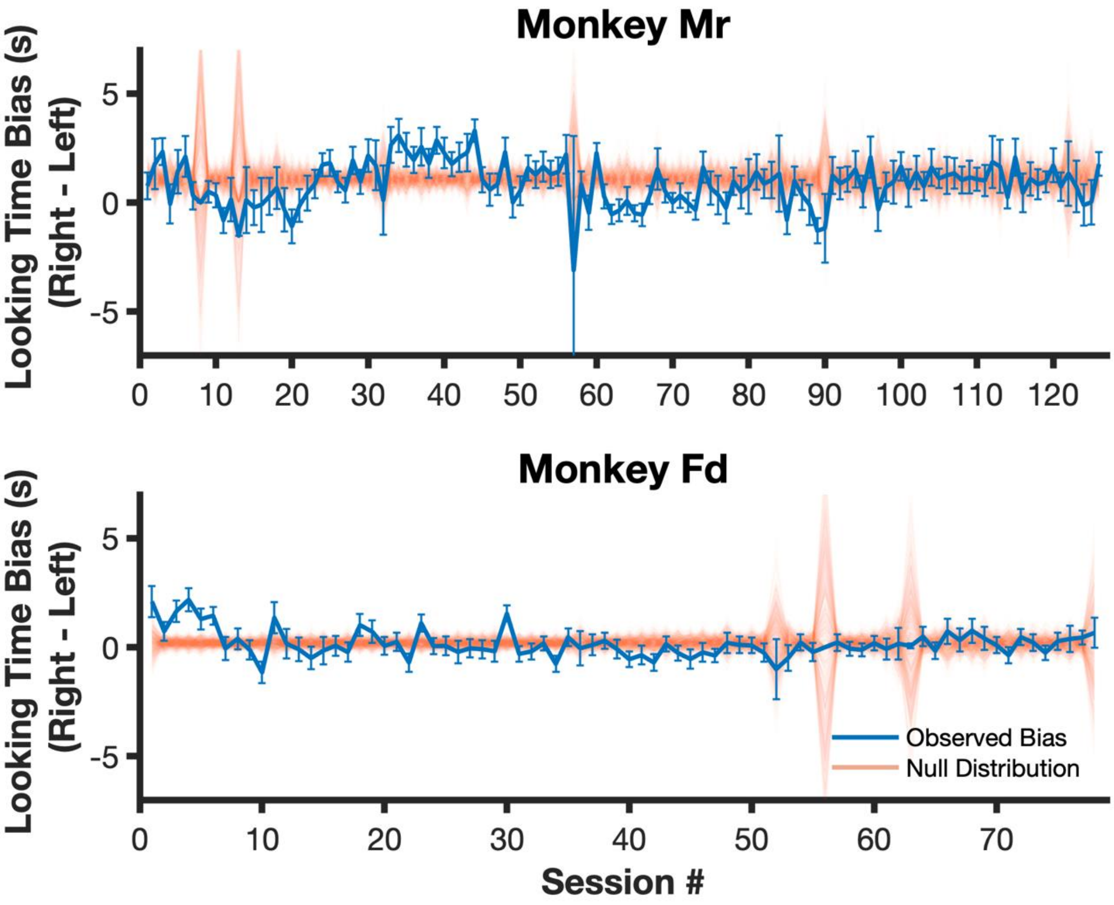

Figure S1: Looking time bias in baseline trials as a function of session number for each animal. The blue lines represent the average looking time difference for each session (in the absence of muscimol), and the error bars indicate the 95% confidence interval. The red transparent lines represent the null distribution estimated by permuting session labels of the data 1000 times.
